# Effect of Named, Accountable GPs on Continuity of Care: Protocol for a Regression Discontinuity Study of a National Policy Change

**DOI:** 10.5334/ijic.2450

**Published:** 2016-03-31

**Authors:** Therese Lloyd, Adam Steventon

**Affiliations:** The Health Foundation, London, UK

**Keywords:** continuity of care, regression discontinuity, electronic health records, Usual Provider of Care index

## Abstract

**Introduction:**

Increasing continuity of care has been identified as a strategy to improve patient outcomes, but previous studies of integrated care have tended to focus on pilot areas, which limit their generalisability and the ability to determine in which contexts integrated care was most successful.

**Objective:**

This study protocol describes a quantitative evaluation of a reform in England that introduced named, accountable general practitioners for all National Health Service (NHS) patients aged 75 years or over. The national contract for general practice services required that named general practitioners offer longitudinal continuity of care within the general practice and be accountable for coordinating care to meet the patient’s healthcare needs.

**Methods:**

This study will apply a regression discontinuity design to pseudonymised electronic medical records from a sample of general practices in England. We will compare outcomes for patients aged just below and above the age of 75 to estimate the effect of named general practitioners and relate these estimated treatment effects to the characteristics of general practices. Outcomes will include a metric relating to continuity of care, namely the Usual Provider of Care Index, and numbers of general practitioner contacts, referrals to specialist care and diagnostic tests.

**Discussion:**

The study illustrates an approach to evaluate national changes aimed at more integrated care using electronic records, which will complement in-depth examination in pilot sites.

## Introduction

There has been a concerted effort in recent years to promote more integrated care in England [1,2,3]. Integrated care is a term often used to describe services that are coordinated around the patient and based around their needs [1,2]. Continuity of care is an important part of this vision, and patient satisfaction is strongly linked to the ability to see a doctor with whom the patient has a personal relationship [4]. However, due to increased specialisation within primary care and the end of ‘personal registration’ with a particular general practitioner in the last 10 years, there is concern that it is becoming more difficult for patients to see their preferred general practitioner [5,6]. In England, 54% of patients have a general practitioner they preferred to see, and this proportion is higher among patients aged 75 and over, at 75% [7,8]. However, among adults with a preferred general practitioner, 31% saw them only some of the time and 8% never or almost never saw them in 2013/14 [9].

The term continuity of care has been used in a variety of ways and can encompass many different ideas [10,11,12,13,14]. One model distinguishes between three related but distinct concepts: longitudinal continuity of care with as few doctors or healthcare professionals as possible; coordinated care, with good communication between professionals and a consistent approach to the management of health needs; and the subjective experience of a caring relationship [12]. There is evidence that the various aspects are associated with positive effects on a range of outcomes, including more appropriate prescribing and higher quality of life [14]. In particular, longitudinal continuity may be an important element in interventions to reduce emergency department visits and unscheduled hospital admissions [14,15,16]. Subjective continuity of care is strongly linked to patient satisfaction [4,10,17], which in turn may be a predictor of compliance with following recommended treatments [14,18]. Although one study reported that patients with a personal relationship with their general practitioner experienced fewer referrals to specialist care than other patients [19], a retrospective cohort study suggested more complex associations between longitudinal continuity and time to referrals and diagnoses for certain cancers [20].

Many evaluative studies of the effect of interventions aimed at greater continuity of care have been conducted within specific localities, which can limit the generalizability of study findings [4,16,17,21,22,23]. However, in 2014, the Department of Health reformed the contract for general practices in England to require that all patients aged 75 or over be appointed a named general practitioner with specific responsibilities for care coordination [24]. This policy provides an opportunity to evaluate the effect of a national initiative on continuity of care and so relates variation in effectiveness to differences in the local context [25]. Although a recurrent concern with ‘top-down’ reforms is that changes might not have the active buy-in of local practitioners [26], with few exceptions [27] these are seldom evaluated. This is an important gap in the literature as contractual reform is one of the few policy-making levers that promise direct impact.

Estimating the effect of interventions aimed at providing more integrated or continuous care is often challenging because studies are typically not randomised [21,28]. Thus, intervention and control groups might differ at baseline and, unless outcomes are accurately adjusted for these differences, inferences can be biased [29]. Studies often use regression adjustment or propensity score matching, but these methods assume that all relevant baseline characteristics have been observed, which may be implausible [30]. Regression discontinuity methods avoid having to make this assumption and are appropriate where eligibility for an intervention changes sharply at a predefined threshold [31]. The key insight is that patients just above and below the threshold should have similar baseline characteristics. Therefore, any discontinuity in the outcome variable at the threshold can be attributed to differences in the treatments received. Regression discontinuity designs have been shown to replicate the results of randomised controlled trials [32] and, although underutilised in health services research, are well established in the economics literature [31,33,34].

The article describes the design of a regression discontinuity study to assess whether the national policy to introduce named accountable general practitioners led to changes in the longitudinal continuity of care and to relate these to the characteristics of general practices.

## Methods

### Reform to the general medical services contract in England

The 2014/15 General Medical Services contract introduced a requirement for general practices in England to offer patients aged 75 or over a named accountable general practitioner, who should provide longitudinal continuity of care within the general practice and be accountable for coordinating care to meet the patient’s health and social care needs. In particular, the named accountable general practitioner should ensure that patients receive all appropriate services and work with relevant associated healthcare and social care professionals to deliver a multidisciplinary care package [24,35]. The contract requires that the practice notify the patient of their named accountable general practitioner, usually by letter, and make a reasonable effort to accommodate patient preferences about the choice of general practitioner. However, patients are not prevented from seeing any other general practitioner in the practice [36]. The 2014/15 GMS contract changes led to a step change (discontinuity) in the percentage of patients receiving a named accountable general practitioner, which increases from around 5% to around 80% near age 75 (Table 1).

**Table 1:** Preliminary analysis of data from Clinical Practice Research Datalink showing that the requisite discontinuity exists in the data.

	Patients aged 70–74 (%)	Patients aged 75–79(%)

Notified of named accountable general practitioner	5420 (5%)	67,770 (80%)
Not notified of named accountable general practitioner	99,830 (95%)	17,161 (20%)
Total	105,250	84,931

*Note:* Based on preliminary analysis of Clinical Practice Research Datalink data. Figures are illustrative as this analysis was based on 2014 data and not all exclusion criteria were applied.

### Study design

**Figure 1:**
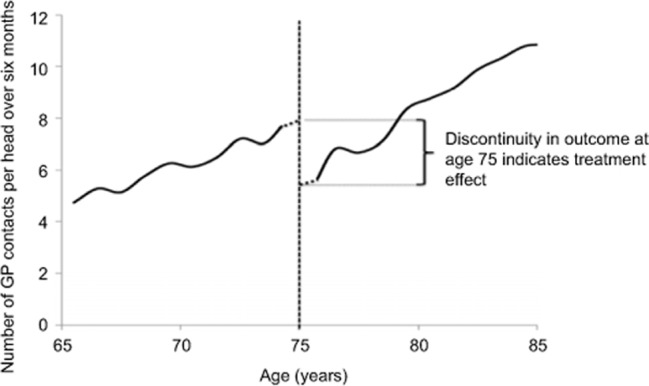
Illustration of regression discontinuity design. Note: Hypothetical data to show the principles of the regression discontinuity design.

Within a regression discontinuity design (illustrated in Figure 1), a step change in outcome at the threshold is attributed to a difference in the treatments received on either side of the threshold. The regression discontinuity design requires much weaker (and hence more plausible) assumptions than other methods, such as regression adjustment, which require that confounders are observed [31,37]. An important assumption underlying all regression discontinuity designs is that, in the absence of an intervention, outcomes will vary smoothly with the running (or treatment determining) variable [31]. Thus, although older people might see their general practitioner more often than younger people, contact rates should not be discontinuous at age 75 in the absence of the introduction of named accountable general practitioners. The assumption seems reasonable and can be validated, for example by ensuring that there are no discontinuities in other covariates at age 75. The standard regression discontinuity design estimates a ‘local treatment effect’, which in our case will apply to patients aged 75. However, preliminary analysis shows that, contrary to the eligibility rules, some patients aged below 75 received a named accountable general practitioner, while some older patients might not (Table 1). As a result, we will apply a ‘fuzzy’ regression discontinuity design, so our treatment effects will additionally apply only to patients who are compliers, i.e. to patients who would receive a named accountable general practitioner if eligible for one, but not otherwise.

### Study cohorts

All general practices in England were mandated to participate in the policy. We will examine data from the electronic medical record of a national sample of general practices that participate in the UK-wide Clinical Practice Research Datalink. This is a validated and widely used research database that contains anonymised, person-level clinical data for all registered patients, linkable to other data sources, such as death records from the Office for National Statistics and Hospital Episode Statistics administrative data [38].

We will study all English general practices that have submitted data to Clinical Practice Research Datalink for the full 2014/15 financial year, excluding practices for which data are identified by Clinical Practice Research Datalink as not being up to research standard or that did not give permission for data linkage. This is expected to result in approximately 200 practices. Within these practices, we will study registered patients born between 1929 and 1949, and thus aged between 65 and 85 in 2014. Patients will be excluded if they have missing year of birth or gender, deregistered within the study period or were identified as at high risk and started admission avoidance care, as part of the Avoiding Unplanned Admissions Enhanced Service. All patients on the Service received named accountable general practitioners irrespective of their age, meaning that a regression discontinuity design is not appropriate for these patients.

### Exposure variable and index dates

For reasons of patient confidentiality, Clinical Practice Research Datalink contains year of birth rather than full date of birth. Therefore, age will be calculated by subtracting the year of birth from 2014, and we will compare patients whose age was in a small bandwidth (interval) above and below 75 years. This bandwidth will be selected using standard cross-validation methods [31].

We will report the proportion of patients in each age group who received the intended treatments. Since the policy ensures that the vast majority of patients who are eligible for a named accountable general practitioner within 2014/15 will have received one by September 2014, treatment receipt will be based on the presence of a recorded named accountable general practitioner within a 6-month period (April 2014 to September 2014), using the Read code prescribed by the General Medical Services contract (67DJ). Patients without a recorded named accountable general practitioner by September 2014 will be regarded as untreated patients, even if they received a named accountable general practitioner subsequently.

All patients will be assigned an index date for the purposes of calculating endpoints and covariates. For patients who are assigned a named accountable general practitioner between April and September 2014, this will be the date at which this was recorded. Patients who are not assigned a named accountable general practitioner during this period will be randomly assigned an index date, such that the distribution of index dates across months is the same as for those patients who did receive one. Thus, all patients will have at least 6 months follow-up within 2014/15, before the policy was extended to all patients irrespective of age [39].

### Study endpoints and covariates

Our primary endpoint will be the number of general practitioner contacts per head over 6 months; this will include all appointment types (e.g. both surgery visits and telephone calls). Secondary endpoints, also calculated over 6 months, will be a measure of longitudinal continuity of care; the number of contacts with general practitioners or practice nurses; numbers of referrals to specialist care; and number of common diagnostic tests (e.g. blood pressure). There are several measures of longitudinal continuity of care, which focus on the concentration, dispersion, distribution or sequence of consultations [12,17]. The Usual Provider of Care index measures the proportion of contacts with the general practitioner who is most commonly seen [17]. The Usual Provider of Care index is widely used [11] and is more easily interpreted than some of the other measures [12]. The Usual Provider of Care index was not chosen as the primary endpoint, as this measure is only defined for the subgroup of study participants with at least two general practitioner contacts during the study period. The theory behind the intervention is such that a change to the number of general practitioner contacts, referrals to specialist care or numbers of diagnostic tests is more likely following an improvement to continuity of care. However, we will analyse these endpoints regardless of the findings for the Usual Provider of Care index, since it is theoretically possible that, for example, the letters led to an increase in enquiries from patients without improvements to continuity.

The following covariates will be calculated for all patients, using data recorded prior to the index date: gender; ethnicity; socioeconomic score (attributed to the patient according to their area of residence, and measured by deciles of the Index of Multiple Deprivation 2010) [40]; number of long-term health conditions recorded prior to the index date; and numbers of general practice contacts, referrals to specialist care, diagnostic tests and hospital discharges in the 6 months prior to the index date. Long-term conditions will be defined in accordance with the Quality and Out-comes Framework. The data quality for the Quality and Outcomes Framework indicators is enhanced as a result of these conditions forming part of the pay-for-performance scheme for general practice [41].

## Statistical methods

Our initial analysis (Table 1) suggests that the proportion of patients receiving a named accountable general practitioner is discontinuous at the threshold, but this will be confirmed on receipt of the data. We will also confirm that, although we would expect a trend in certain covariates across age, there are no discontinuities in these covariates at age 75. This comparison will be done graphically and include all patients, regardless of actual treatment assignment. We do not expect such discontinuities to occur, but if they do exist, this would lead us to question the validity of our study design.

As mentioned previously, the proportion of patients receiving a named accountable general practitioner is unlikely to jump from 0 to 100% at age 75. It will therefore be appropriate to apply a fuzzy regression discontinuity design [31], by fitting four regression models. Two models will relate the outcome to a polynomial function of age: one model will be fitted for people whose age lies within the bandwidth below the threshold and the other model for people within the bandwidth above the threshold. The final two models have treatment assignment as their dependent variable rather than outcome. All models will use ordinary least squares regression and include patients irrespective of their actual treatment assignment. We will calculate the estimated treatment effect as the difference between the predictions of the two outcome models at age 75, divided by the difference between the predictions of the two treatment models at age 75.

The analysis will have regard to the rounded nature of the running variable (i.e. age), resulting from the use of year of birth rather than full date of birth. This will exclude the use of very small bandwidths, and so we will use a larger interval spanning several years and model any trend in the endpoints by age using parametric approaches based on polynomial functions [42,43]. In the absence of information on full date of birth, it will not be possible to establish for a given patient born in 1939 what date they turned 75, and therefore which side of the threshold the patient is at the index date. We will remove the risk of misclassification by excluding all patients born in 1939 when fitting the regression models and making a small extrapolation around age 75 [42]. A final consideration is that, when the running variable is only observed at discrete points, discretisation bias can occur because a change in slope close to the cut-off might be mistaken for a treatment effect when in fact it is due to the ‘natural’ change in outcomes with age. We will correct for this bias using a method that is based on the moments of the age distribution [42].

We will conduct a series of additional specification tests to check the validity of the study design [31]. First, we will repeat the estimation procedure at non-discontinuity points, to confirm a treatment effect of zero. Second, we will confirm that the estimated treatment effect is not sensitive to the choice of bandwidth or order of polynomial. Third, we will repeat the analysis when including covariates in the regression models. Although it is not necessary to include covariates in regression discontinuity analyses, including them may improve precision [37,42].

### Subgroup analyses

We expect that general practices will implement named accountable general practitioners in various ways and that the effectiveness of the intervention may depend on factors related to the local context in which the general practices operate [25]. Therefore, we will characterise general practices according to: setting (urban/rural, based on the RUC2011 classification at small area level) [44], average socioeconomic deprivation score of their patients, number of general practitioners within the practice and practice list size. We will also include the average number of patients per full-time equivalent general practitioner. We will estimate treatment effects separately for each general practice and then test the relationship between these treatment effects and general practice characteristics using a practice-level, linear regression model [45].

### Required sample size

We believe that a 5% change in the number of general practice contacts would represent a meaningful difference to general practice workload, since this represents approximately one extra appointment per working day for an average general practitioner [46]. Assuming an average of 5 general practitioner contacts per 6-month period in the absence of a named accountable general practitioner [47], and a standard deviation of 6 contacts [47,48], we will need 24,212 patients in each treatment group, at 90% power and two-sided p-value of 0.05. Although the precise statistical power of the study will depend on the bandwidth, the Clinical Practice Research Datalink database will provide more than the required number of patients (Table 1) [37].

## Discussion

### Contribution of the study

Both policy-makers and patients believe that general practitioners are best placed to coordinate their care [13,24,49], but many patients cannot see their preferred doctor [9]. The current study will evaluate the introduction of a named accountable general practitioner for each patient aged 75 or older and the requirement to communicate to patients their specific responsibilities for coordinating health and social care. As well as assessing impacts on various metrics relating to continuity of care, including the Usual Provider of Care index, we will investigate the variability in the effectiveness of the policy between general practices with different characteristics.

### Strengths and limitations

Four characteristics of this study distinguish it from existing work in this area. First, this study will examine a national policy intervention to integrate care, as opposed to particular local initiatives to encourage more integrated services. The nature of national initiatives means that the prescribed intervention might not suit all local contexts and there may be limited local buy-in to change, potentially limiting effectiveness. However, contracts are a potentially important method of encouraging more continuity of care, and we are not aware of previous studies that have investigated the effectiveness of such an approach.

Second, previous studies have examined integrated care within pilot areas [21], but the current study will use a large database to examine a national sample of general practices, increasing the generalisability of its results. The Clinical Practice Research Datalink database is broadly representative of the UK population [41], though our sample will be limited to those that have consented to participate in the Clinical Practice Research Datalink linkage scheme and in addition have submitted data to Clinical Practice Research Datalink in a timely fashion. While it will not be possible for us to document the intervention as closely as pilot studies, we will investigate whether the effectiveness of the policy varies according to local characteristics. If some areas perform better than others, then future studies could use qualitative methods to construct theories for why these areas saw more promising results [50]. The current study is an example of a relatively new approach that applies surveillance methods to national databases to identify areas of good practice, rather than starting with prospective studies of a small number of selected pilots, which may not show the anticipated returns [51].

Third, the large sample size means that we will have statistical power to detect small effects, such as a 5% change in the number of general practitioner contacts. This is important because many previous studies of out-of-hospital interventions have assumed relatively large effect sizes when estimating the required sample size, for example that emergency hospital admissions could be reduced by 15% within 12 months [52,53]. Where such studies fail to detect changes, it is unclear whether this is due to an ineffective intervention or simply that the study was underpowered to detect small yet meaningful effects. Moreover, where interventions are implemented haphazardly or with limited fidelity to the intervention design, this might reduce the power of the study, underscoring the need to assume conservative effect sizes when estimating the required sample size.

Finally, many previous studies of integrated care interventions have used study designs that are susceptible to con-founding arising from differences in unobserved or observed patient characteristics [21,28]. In the current study, the use of a regression discontinuity design will largely avoid these concerns.

One limitation of this study is that the lack of full information on date of birth means that our estimates are dependent on model specification [33], though we will test the sensitivity of our findings. Also, the estimated treatment effects will only apply to individuals aged close to 75 years, while younger or older groups might see larger or smaller treatment effects. Our findings will also apply to the subset of patients who are compliers and are not in the top 2% risk category. By excluding the 2% of the population most at risk, we are omitting some of the patients most in need of integrated care. However, a more intensive intervention is currently being targeted at this group [54], and this intervention needs to be evaluated as a separate study, something we might do at a later stage.

This study will examine four endpoints that could plausibly be affected by named accountable general practitioners, namely general practitioner contact rates, longitudinal continuity of care, referrals to specialist care and numbers of diagnostic tests. It is possible that improvements in these endpoints may take longer than 6 months to materialise but, to the extent that this is due to poor implementation, the subgroup analysis may be informative. Moreover, while the study will examine longitudinal continuity of care, it will not examine other aspects of continuity, such as informational or management continuity [12,13], nor the patient experience of care.

We cannot determine whether the particular general practitioner contacts, diagnostic tests and referrals to specialist care were appropriate. Although continuity of care indices have been successfully applied to Clinical Practice Research Datalink data in the past [20], difficulties in analysing longitudinal data have been highlighted [12]. For example, although a staff identifier is entered onto the computer system, practices may use shared login details, particularly for locum doctors, which would affect the Usual Provider of Care index.

We will not examine impacts on hospital admissions or disease control beyond the frequency of diagnostic tests, as these are unlikely to be affected by the policy within 6 months. Our endpoints are likely to be on the causal pathway for better disease control and reduced admissions, and so our findings will indicate whether investigation of additional metrics is warranted. The limitations of the data mean that it is not possible to examine impacts on social care utilisation or access to primary care.

## Conclusions

This study will analyse a national policy initiative aimed at promoting integrated care through increasing the continuity of care and prompting communication about accountability for care coordination with patients. It will use surveillance of national data to identify sites demonstrating promising outcomes associated with a particular integrated care initiative. This study could complement existing approaches aimed at in-depth examination of particular pilot sites, while removing the threats to confounding from treatment selection associated with many cohort studies.

### Research governance

The Independent Scientific Advisory Committee for MHRA Database Research has approved this study (reference: 15_070R). Ethical review was not required as this is a retrospective study of pseudonymised, routinely collected data.

## Competing Interests

The authors declare that they have no competing interests.
